# Exploring healthcare professionals’ motivation to attend two postgraduate education programs at the University of Bern in Switzerland: A qualitative interview study

**DOI:** 10.3205/zma001761

**Published:** 2025-06-16

**Authors:** Melanie De la Rosa, Felix M. Schmitz, Joana Berger-Estilita, Ara Tekian, Sissel Guttormsen

**Affiliations:** 1 University of Bern, Institute of Medical Education, Bern, Switzerland; 2 Hirslanden Hospital Group, Salem Spital, Institute of Anesthesiology and Intensive Care, Bern, Switzerland; 3 University of Illinois at Chicago, Department of Medical Education, Chicago, USA

**Keywords:** motivation, student satisfaction, higher education, post-graduate education, healthcare professionals, program development

## Abstract

**Introduction:**

Postgraduate education programs for healthcare professionals have seen rising demand due to socio-demographic shifts, digitalisation, and evolving healthcare models. This trend underscores the need for providers to offer high-quality, innovative teaching and learning opportunities that align with participants’ needs and interests. Understanding the motivations behind enrolment is essential for tailoring curricula and didactical methods effectively. This study explores key motivational factors influencing healthcare professionals’ decisions to join postgraduate education programs. The findings aim to inform curriculum planning to ensure that programs better meet the specific needs and interests of their learners.

**Methods:**

We conducted a qualitative study with N=23 semi-structured interviews with 13 students from the Master’s program in Medical Education (MAS) and 10 with students from the Certificate of Advanced Studies in Palliative Care (CAS). The interviews were thematically analysed to identify common and differentiating motivational factors, with coding conducted independently by two researchers to enhance reliability.

**Results:**

This study identified both general and program-specific motivational factors among MAS and CAS participants. MAS participants frequently mentioned career development, professional advancement, and empowerment as key motivations. CAS participants, on the other hand, placed greater emphasis on content-specific motivations. For both groups, local proximity and the education format played a crucial role in their decision to enrol in these programs. In addition, seven implications for program planning were identified to enhance the alignment of postgraduate education with participants’ needs.

**Discussion:**

With the method of qualitative analysis and the focus on post-graduate program-participants’ needs and interests, new insights on motivation to attend postgraduate education were gained. The significance of these findings is supported by existing literature. Consequently, these results offer valuable implications for the planning and development of postgraduate education programs.

## Zusammenfassung

**Einleitung:**

Die Nachfrage nach postgradualen Studiengängen für Fachpersonen im Gesundheitswesen steigt kontinuierlich an. Grund dafür sind soziodemografische Veränderungen, fortschreitende Digitalisierung sowie die Entwicklung neuer Gesundheitsmodelle. Der Trend verdeutlicht die Notwendigkeit für Hochschulen, qualitativ hochwertige und innovative Lehr- und Lernangebote zu schaffen, die auf die Bedürfnisse und Interessen der Teilnehmenden ausgerichtet sind. Um Curricula und didaktische Methoden zielgerichtet gestalten zu können, ist ein vertieftes Verständnis der Motivation zur Teilnahme an solchen postgradualen Studiengängen von wesentlicher Bedeutung. Die vorliegende Studie untersucht die zentralen Motivationsfaktoren, die Fachpersonen im Gesundheitswesen zur Teilnahme an postgradualen Studiengängen bewegen. Die Ergebnisse der Untersuchung sollen einen Beitrag zur bedarfsgerechten Entwicklung von Curricula leisten.

**Methoden:**

Im Rahmen dieser qualitativen Studie wurden N=23 halbstrukturierte Interviews mit 13 Studierenden des Master of Medical Education (MAS) und 10 Studierenden des Certificate of Advanced Studies in Palliative Care (CAS) an der Universität Bern geführt. Die thematische Analyse der Interviews hatte zum Ziel, sowohl gemeinsame als auch differenzierende Motivationsfaktoren der Teilnehmenden zu identifizieren. Zur Erhöhung der Reliabilität wurde die Studie durch zwei unabhängig voneinander arbeitende Forscher kodiert.

**Ergebnisse:**

Die Studie identifizierte bei den MAS- und CAS-Teilnehmenden sowohl allgemeine als auch programmspezifische Motivationsfaktoren. Während MAS-Teilnehmende häufig Karriereentwicklung, berufliche Weiterentwicklung und Empowerment als primäre Motivationen nannten, legten CAS-Studierende häufiger inhaltlich-thematische Beweggründe dar. Für beide Gruppen erwiesen sich zudem die Nähe zum Kursort sowie das Unterrichtsformat als ausschlaggebende Faktoren für die Teilnahmeentscheidung. Insgesamt wurden sieben Implikationen abgeleitet, um postgraduale Studiengänge besser auf die Bedürfnisse der Teilnehmenden auszurichten.

**Diskussion:**

Durch die Anwendung qualitativer Analysemethoden und die Fokussierung auf die Bedürfnisse und Interessen der Teilnehmenden, an postgradualen Studiengängen teilzunehmen, konnten neue Einsichten in deren Motivation gewonnen werden. Die Relevanz der Ergebnisse wird durch die bestehende Literatur gestützt. Auf dieser Grundlage lassen sich praxisrelevante Empfehlungen für die Planung und Weiterentwicklung von postgradualen Studiengängen ableiten.

## 1. Introduction

The need for postgraduate education programs for healthcare professionals has increased in the last decade. Both the variety of programs and the number of attendees is increasing [1], [2], [3], [4]. Possible reasons for the increased awareness of healthcare related postgraduate education programs are socio-demographic shifts, a broadening meaning of health, increased health literacy resulting in empowered patients, the digitalisation in healthcare and the emergence of new models of care [5]. 

These trends not only underscore the increasing demand for advanced qualifications but also directly shape the motivations and needs of healthcare professionals. An aging population necessitates specialized training in fields such as geriatrics and chronic disease management. Simultaneously, the evolving definition of health toward holistic approaches prompts physicians to seek further education that equally addresses physical, psychological, and social aspects of patient care. The digitalisation of healthcare further amplifies this educational need, as proficiency with electronic health records, telemedicine, and modern diagnostic tools requires specialized skills. These challenges motivate healthcare professionals to seek targeted programs that enable them to meet changing demands effectively. The medical domain is complex and dynamic; therefore, the relevance of postgraduate education programs is increasing [6].

Higher Education Institutions (HEIs) providing postgraduate education programs face the challenge of the increasing number of competing programs [6]. To attract learners to HEI’s post-graduate education programs, they must provide best-practice and up-to-date learning contents and didactical methods that are aligned with the changing needs and interests [7] – as expressed by the participants motivation to sign up. 

For program developers to compete these challenges, it is strongly recommended to address the participants’ needs and interests. Both the Swiss Higher Education Council for Accreditation in Higher Education (HFKG) and the Swissuniversity (the consortium of all Swiss universities) in cooperation with the Body for accreditation and quality assurance of Swiss Universities (OAQ) [8], [9], emphasize a clear target group orientation. To uncover immanent implications for future postgraduate education programs development, the learners’ particular needs and interests must be explored at first place.

While previous research has examined general motivational factors for professional development in the healthcare sector, a gap exists concerning the specific motivations of participants in Swiss postgraduate educational programs, particularly those at the University of Bern.

The present study aims to bridge this gap by identifying the essential motivational factors of participants in these programs. Moreover, our broader goal is to derive recommendations for curriculum planning of healthcare related postgraduate education programs.

## 2. Background

In this context, the “motivation concept” is multifaceted and needs some clarification. One perspective is the *general motivation* to enrol in a postgraduate education program. Examining this perspective, an early motivation model is offered by Houle [10]. He relates motivation to learning orientation and identifies three related factors: goal-, activity-, and learning orientation. “goal orientation” addresses the need to learn to fulfil a clear purpose and get ahead in career or life. “Activity orientation” refers to the learners’ need for social and intellectual stimulation. Finally, “learning orientation” addresses the passion for learning. Boshier [11], [12] tested and further developed Houle’s model; for the optimal measure of participation motivation in adult education, he constructed the “Education Participation Scale” (EPS) [11], [12], [13]. Building on this research, Garst and Ried [14] built on one of Boshier’s models comprising six motivation factors and modified them with data from healthcare professionals:

1. interpersonal relations (e.g., take part in activities, share an interest with others),
2. compliance with external influence (e.g., professional obligation, recommendation of someone),
3. professional advancement (e.g., keep up with competition, higher status),
4. escape from routine (e.g., get away from responsibilities, relief from boredom),
5. competency-related curiosity (e.g., intellectual curiosity, practical benefit), and
6. community service (e.g., become effective as a citizen).

This model is particularly relevant because it addresses both intrinsic and extrinsic motivation perspectives, which also have been reflected in Academic Motivation Scale (AMS/EME) described by Vallerand [15]. Further models exist to describe the motivation for educational programs, e.g. the Jefferson “Scale of Physicians Learning” [16], [17] or the “Hennessy Hicks Training Needs Analysis Tool” (HHTNAT) [18]. In both models, similar motivation factors to those from the EPS have been described. Furthermore, the six motivation factors of the EPS could be verified in studies from 9 different regions, including Canada, US and Europe [19], [20], [21], [22], [23], [24], [25].

Next to the models described above, we could identify several studies, addressed below, describing the *motivational factors for participating in a particular program*. Some of these studies combine particular motivation factors with general ones. Henry and Basile [26], for example, identified factors that affect the decision to enrol in formal adult education. Their study mentioned general reasons for enrolling (e.g., meeting new people, general interest, job-related) and specific factors (e.g., location, timing, course attributes). In the PRISM-T Model, Stein and Wanstreet [27] also combine general motivation factors, like the possibility for intellectual, personal and career opportunities, with specific decision factors, like institutional support and academic reputation. The T in the model stands for the overarching time factor [27]. Based on the literature reviewed, we can identify and emphasize seven factors that describe motivation factors to attend a particular program [20], [21], [22], [26], [27], [28], [29], [30], [31], [32], [33], [34], [35], [36], [37], [38]:

1. practical factors (e.g., time management),
2. factors related to contents (e.g., relevant clinical topic),
3. education format (e.g., interactive program),
4. network building (e.g., social aspect of learning),
5. reputation (e.g., sponsoring organization),
6. recommendation (e.g., decision support tools),
7. faculty (e.g. reputation of teachers).

Based on our preliminary reflections, the overall aim of the present study is to investigate relevant motivation factors for participants to attend healthcare-related postgraduate education programs (both general and particular). The key idea of generating insights about students’ motivation, is that it should have impact on program planning. 

The following research questions (RQ) reflect our aims:

- *RQ1: What motivates healthcare professionals to participate in postgraduate education programs in general?*
- *RQ2: What motivation factors influence the decision of health care professionals to attend a particular postgraduate education program? *
- *RQ3: What implications for future program planning can be derived from the identified motivation factors? *

## 3. Method

### 3.1. Developing interview guideline 

The research questions were addressed using a qualitative approach with semi-structured interviews with participants of two postgraduate education programs. We developed an interview guideline inspired by the 5-steps procedure described by Gideon [39]: 

#### Step 1: Review relevant literature

In our literature review, we identified the EPS, as proposed by Garst and Ried [14], as the conceptual framework for addressing the first research question. For the second research question, we considered multiple factors that were evaluated in the literature review.

##### Step 2: Write question items

Based on the results from step 1, we drafted a set of initial questions addressing the identified factors. We developed open questions to obtain a comprehensive picture of answers and not to impose any answers. This means that the interview questions are not directly linked to the individual factors respectively but are intended to induce responses that address the initially identified theoretical factors. The process resulted in an initial large set of questions, which were revised in several steps by the research team. 

##### Step 3: Structuring and elaborating the question route

The question route was structured in three blocks: introduction, main part and the final part. The introduction part was about creating a positive interview atmosphere. After a short welcome, the study topic was introduced, and the interview process explained. In the introduction, the first “icebreaker” questions were asked. The main part of the questionnaire contained two sub-question blocks addressing RQ1 and RQ2, respectively. In each of the two-sub-question blocks, first, an open question close to the respective research question was prepared. Depending on the answers, nine to ten further predefined questions supported the interviewer in prompting on further aspects. In the final part of the interview, the interviewee was thanked, space for questions was given, and the next steps were explained.

##### Step 4: Pilot testing

The interview was piloted with three participants from other MAS/CAS programs not included in this study. This allowed us to check the comprehensibility of the questionnaire, the time needed, and the acceptance of providing answers. After this pilot phase, we added, deleted, and reconstructed questions. 

##### Step 5: Finalise and administer questionnaire

In the last step, we finalised the question route. Formulations, grammar, and specific wording were finalised. The final question route consisted of 24 items: 3, 11, and 10 for the three parts, respectively (see attachment 1 ). Recognising that the interviewer’s role may affect participants’ responses, we adopted an open-ended questioning style and encouraged reflection to minimize leading or suggestive questions.

### 3.2. Recruiting participants and conducting the interviews 

We identified two postgraduate education programs offered to interprofessional healthcare professionals fitting the targeted programs: the Master of Advanced Studies program (MAS), “Master of Medical Education” (MME) and the Certificate of Advances Sciences program (CAS), “Palliative Care” (PallCare), both offered by the University of Bern. The programs and participants were selected through convenience sampling [40], based on three criteria: active engagement in clinical or healthcare educational roles, current enrolment or recent completion of the MAS or CAS programs, and willingness to participate. This sampling method was chosen due to the limited number of relevant postgraduate programs available, facilitating a practical and efficient data collection process. 

The two-year, part time MAS in Medical Education primarily attracts professionals such as physicians and clinical teachers who are seeking to improve their skills in medical teaching, curriculum design, and educational leadership. This program is offered every two years by the University of Bern and managed by the Institute for Medical Education at the medical Faculty in Bern, is delivered in English, offered to an international group of students, and accommodates up to 25 participants per intake [41].

The CAS in Palliative Care is a 1.5-year, part-time program, offered by the University of Bern and managed by the Centre for Palliative Care at the Insel University Hospital in Bern. Designed for a range of healthcare professionals, including doctors, nurses, and social workers, this interprofessionally oriented program aims to enhance expertise in providing holistic and compassionate care for patients with life-limiting illnesses. While the program is open to various professions, the majority of students come from the nursing sciences. The program commences every two years, is delivered in German, is intended for individuals living in Switzerland, and accommodates up to 25 participants per intake [42].

We conducted 13 interviews with participants of the MAS (all participants in the first year), and 10 interviews with participants of the CAS (5 interviews with second-year students and 5 interviews with recently graduated participants). The interviews were conducted by the first author of this article. Interviewees were added to the sample until saturation was reached [43]. The interviews lasted, on average, 60 minutes and took place online via Zoom. The interviews were all conducted in German language. Statements in this article are translations of these German text passages. All participants signed an informed consent from for the recording, transcription, and later use of the material. The Bern cantonal ethics committee granted ethical approval (BASEC-Nr: Req-2022-00555). 

### 3.3. Qualitative content analysis

Each of the 23 interviews was transcribed according to a protocol of intelligent verbatim transcription [44]. The transcriptions were analysed using the “Content Structuring Qualitative Content Analysis” from Kuckartz [45]. 

We started with an initial text work, reading and rereading the transcribed interviews to become familiar with the contents. In the next step, main categories were developed using the tool NVivo [46]. Following Kuckartz’ consensual coding-technique [45], parts of the contents were coded by an additional researcher and later discussed to reach consensus. The first coding was done in a combination of deductive and inductive analysis. Deductive themes are based on the categories identified from the literature review (general and particular motivation). New themes were identified inductively. 

After this first consensus coding run, all data were coded again using the main themes developed both inductively and deductively. In the next step, the two researchers inductively coded subcategories within all main categories. The list of subcategories that now resulted was discussed and summarised by a team of 3 researchers. In this phase, we also discussed and decided in a consensus-based manner whether identified subcategories fit suitably to the given main categories. After the subcategories had been defined, a second coding process took place to verify, correct and finalise the subcategorisation. 

To avoid bias in over- or underrepresenting themes, we decided to report each theme based on the number of participants who mentioned it, rather than the number of mentions per participant. Themes were included only if mentioned at least five times across the entire dataset. This threshold ensures that the identified themes are recurrent and reflect their significance across multiple participants, thereby increasing the reliability of the findings.

### 3.4. Procedure of deriving implications for future program planning 

Based on the results of the qualitative content analysis, the first author identified themes and mentions which likely could serve as implications for program developers. A team of experts collaboratively examined the suggestions in a workshop validating their implications for program planning. 

## 4. Results

### 4.1. Descriptive data

In total, seven men (*n_MAS_*=5, *n_CAS_*=2) and 16 women (*n_MAS_*=8, *n_CAS_*=8) aged between 26 and 62 years (M=43) participated in the interviews. Eleven participants held an employee position (*n_MAS_*=3, *n_CAS_*=8), and eleven had a leadership position (*n_MAS_*=10, *n_CAS_*=1). In total, 16 physicians (*n_MAS_*=11, *n_CAS_*=5), 3 nurses (*n_CAS_*=3), 1 social worker (*n_CAS_*=1), 1 lawyer (*n_CAS_*=1), 1 person with an MA in intercultural studies (*n_MAS_*=1) and 1 with a biomedical background (*n_MAS_*=1) were interviewed. 

Table 1 (Tab. 1) provides an overview of the identified deductive and inductive motivation themes related to RQ1 and RQ2. 

### 4.2. General motivation themes to participate in postgraduate education programs 

An overview of the identified general motivation themes, the most mentioned subthemes and sample quotes of the interviewees that illustrate the results, is given in table 2 (Tab. 2). Below, we highlight the most important themes from attachment 2 and comment of similarities and/or differences between the groups. 

The most mentioned theme for attending continuing education for both groups is: “competency-related curiosity” (*n_MAS_*=13, *n_CAS_*=9). The most frequently mentioned subtheme for both groups is “interest in topic” (*n_MAS_*=13, *n_CAS_*=9). Differences between the study programs, with a difference of seven mentions, lies in the subtheme “personal development” (*n_MAS_*=11,* n_CAS_*=4), which was more prominent in the MAS group. 

The theme “professional advancement” is often mentioned (*n_MAS_*=13, *n_CAS_*=8). The subthemes “adapting to workplace changes” and “career springboard” (*n_MAS_*=9, *n_CAS_*=6) stand out in both groups (*n_MAS_*=12, *n_CAS_*=6). 

The theme “compliance with external influence” was renamed to “external influence” (*n_MAS_*=13, *n_CAS_*=8) to better fit the identified subthemes. The subtheme “circumstances” is the most frequently mentioned subtheme within this theme among CAS participants (*n_CAS_*=5). The greatest difference between the study programs, with a difference of 8 mentions, lies in the subtheme “manager as initiator” (*n_MAS_*=9, *n_CAS_*=1).

The theme “escape from routine”, was excluded from our results due to the few mentions. Instead, we identified the inductive theme “cognitive stimulation” (*n_MAS_*=11, *n_CAS_*=7). Inside this theme, the subtheme “stimulation” was mentioned more often in the group of the the MAS participants (*n_MAS_*=7, *n_CAS_*=3). For both MAS and CAS participants, the subtheme “fun and joy in learning” is relevant (*n_MAS_*=5, CAS=4).

An inductively newly identified theme is “empowerment” (*n_MAS_*=11, *n_CAS_*=6). The most frequently mentioned subtheme here for both groups is “evoking changes” (*n_MAS_*=10,* n_CAS_*=5). The subtheme “carrying the title” has a higher importance for MAS participants than for CAS participants (*n_MAS_*=6, *n_CAS_*=3)

The theme “interpersonal relations” was renamed to “networking” (*n_MAS_*=10, *n_CAS_*=6) as all subthemes indicate this direction. Whereas the participants of the MAS focused on the subthemes “build a network” (*n_MAS_*=9) and “inspiration through exchange” (*n_MAS_*=9), the CAS participants named mostly “inspiration through exchange” (*n_CAS_*=5).

The theme “community service” was renamed to “social responsibility” (*n_MAS_*=4, *n_CAS_*=3). For both groups this theme has the lowest influence as motivation theme to participate in postgraduate education programs. 

In sum, the main themes of general motivation in attachment 2 show converging and diverging relevance and weighting for the groups. The major group differences are found in the inductive subthemes. Two factors from the literature were directly identified in our data, three were renamed for a more accurate representation, and one was not identified at all. Two entirely new inductive themes emerged. As a result, the study concludes with seven themes that, in certain instances, deviate from the initially proposed theoretical factors and introduces additional subthemes for each.

### 4.3. Motivation themes to join a particular postgraduate education program 

To address the second research question, we explored the identified motivation themes related to participation in a particular program, in the order of mentions. In this case, we could identify all the deductive themes in our data; all the subthemes were inductive. Attachment 3 shows the most frequently mentioned subthemes for each theme across both groups, alongside sample quotes that illustrate these subthemes. We included those themes that are mentioned at least five times in total. Below, we provide descriptions of the themes and the two most mentioned subthemes.

The most frequently mentioned theme regarding the motivation to participate in a particular postgraduate education program is “practical factors” (*n_MAS_*=13, *n_CAS_*=10). The most frequently mentioned subtheme within this main theme for both groups is “proximity” to place of learning (*n_MAS_*=11, *n_CAS_*=9), and the second most mentioned subtheme is “support from employer” (*n_MAS_*=7, *n_CAS_*=4).

“Education format” (*n_MAS_*=13, *n_CAS_*=9) is the second most mentioned theme. The most frequently mentioned subtheme in both groups is “structure and organization” (*n_MAS_*=13, *n_CAS_*=7). For the MAS participants, this subtheme has even more importance as it is the most mentioned subtheme of all subthemes. The subtheme “method” has less relevance for both groups (*n_MAS_*=4, *n_CAS_*=5).

“Factors related to contents” was frequently mentioned as a reason to attend the particular program in both groups (*n_MAS_*=12, *n_CAS_*=8). A bigger difference between the groups was found in the subtheme “practical relevance”. While it is highly relevant for the MAS participants (*n_MAS_*=10), it is not that important for the CAS participants (*n_CAS_*=2). In the CAS group the subtheme “chosen topics” stands out with six mentions.

“Faculty”, in the meaning of teachers is also a frequently cited reason in both groups (*n_MAS_*=12, *n_CAS_*=8). The subtheme “experts competency” stands out in both groups (*n_MAS_*=9, *n_CAS_*=8). The subtheme “international perspectives” is only prominent in the group of MAS participants (*n_MAS_*=5, *n_CAS_*=0).

The theme “reputation” was mentioned as a reason to attend the program in both groups (*n_MAS_*=12, *n_CAS_*=6). The subtheme “reputation of the organization” (*n_MAS_*=10, *n_CAS_*=5) was mentioned slightly more often than the “reputation of the program” (*n_MAS_*=9, *n_CAS_*=3).

Another reason for choosing a particular program is “recommendation” (*n_MAS_*=10, *n_CAS_*=5). This theme is more significant for MAS participants. Also, the subthemes “influence of peers” (*n_MAS_*=9, *n_CAS_*=3) and “influence of leadership/role model” (*n_MAS_*=6, *n_CAS_*=3) were mentioned more frequently among the MAS than among CAS participants. 

“Network building” was also mentioned as a reason to attend the particular program (*n_MAS_*=6, *n_CAS_*=4). This main theme does not have any subthemes. 

### 4.4. Implications for future program planning 

In validating RQ 3, and deriving the seven practice implications, the data generated in RQ1 and RQ2 were used. The implications are formulated as heuristic guidelines for program developers. Statements illustrating these implications are shown in table 2 (Tab. 2). 

## 5. Discussion

### 5.1. General and particular motivation to participate in postgraduate education programs 

This study shed new light on both general motivation to attend postgraduate education programs and the motivation to attend a particular program. The findings, summarized in attachment 2 and attachment 3 , underline the relevance of the theoretically derived factors and suggest relevant inductive themes: the inductive themes Empowerment und cognitive stimulation. The relevance of both factors for health professionals is confirmed in the literature by the concept of empowerment [47] and the lifelong learning theory [48].

Possible explanations for the importance of factors, as well as the similarities and differences between general motivation and motivation for a particular training program, are closely related. Hence, we will integrate the findings from the two research questions and discuss the specifics of each program.

For MAS students, who often occupy or aspire to leadership positions, there is a pronounced emphasis on factors around the topic *career development*. Factors such as “competency-related curiosity”, “professional advancement”, “cognitive stimulation”, and “empowerment” were frequently mentioned. Also the often mentioned subthemes “personal development”, “career springboard” or “chosen topics” reflect the pressures of staying abreast of healthcare trends [5]. “Empowerment” and the subtheme “evoking changes” are particularly crucial. The participants wish to be empowered to negotiate with superiors or to influence future events and developments. It is of great importance for healthcare professionals to influence changes that may affect their work and the health of their patients [49]. The particular motivation subtheme “factors related to contents”, with subthemes such as “chosen topics” and “evidence-based” are important for imposing changes in a medical academic context. The general motivation theme “external influence” and the subtheme “manager as initiator” underscore the structured and institutionalized nature of career development in this sector. The theme of “networking” and the subtheme “build a network” carries the same significance. The participants aim to establish regional contacts, exchange ideas, and potentially foster collaborations and partnerships. Health professionals recognise these benefits to their professional development [50], [51]. Additionally, the role of “reputation” and “recommendations” in program selection has relevance for MAS participants. The longer duration and higher cost of the MAS program necessitate a more calculated decision influenced by external advice and the credibility of the program.

Conversely, from the interviewees in the CAS program, all themes remain relevant, but there is a substantial focus on “competency-related curiosity” and “professional advancement”. The subtheme “interest in topic” and the particular motivation theme “factors related to content” and the subtheme “chosen topics” describe this focus. This thematic orientation could be related to the speciality of the topic “palliative care”. In palliative care there is a particular need to consider the patient's physical, psychological, spiritual, and social needs. Patients are considered from a holistic point of view as essential in the context of near-death or critical life phases [52]. 

Noteworthy for both groups is the importance of “practical factors” and the “education format” such as “proximity” and the “structure and organization” of the program. Both programs cater to working professionals with demanding jobs, families, and social lives. Participants select a program based on its integration into their daily lives and organizational applicability.

In sum, both programs share similar primary themes across target groups, indicating that the core motivational factors are broadly relevant. The diversity of our participants, hailing from various healthcare professions, suggests that these motivational factors may also be pertinent to other disciplines within the healthcare sector. This versatility is supported by literature discussed in our theoretical framework. The analysis, however, shows that similar themes in the two sections, “general-” and “particular”-motivation, have different subthemes, impacts, and meanings. The motivation to attend a postgraduate education program, either general or particular, is related to the participants’ position, the topic and the program profile. These insights are valuable for developing academic offerings that align with the needs of healthcare professionals, thereby, enabling HEIs to offer high-quality, innovative teaching and learning opportunities, which in turn bolsters their competitiveness in the educational landscape.

### 5.2. Implications for future program planning 

In validating RQ3 the data gathered for the analysis of RQ1 and RQ2 were re-examined with a focus on generating implications. This process resulted in seven practice implications, which are validated below. We emphasize that qualitative research does not provide indisputable evidence; rather, the implications drawn from qualitative data are suggestive and offer valuable directions for future research and reflection. Consequently, these implications are formulated as heuristic guidelines for program developers, illustrated by the quotes outlined in table 2 (Tab. 2).

Furthermore, it is crucial to recognise that if these motivational factors, outlined in the following implications, are not adequately addressed, the overall student satisfaction and learning experience may suffer. Student satisfaction plays a pivotal role in the competitiveness of postgraduate education programs. Research indicates [53] that institutions with higher levels of student satisfaction tend to be more competitive, as they attract and retain students more effectively.

#### Implication 1: Regularly and critically reflect on the participants’ professional needs, preferred topics, and professional and social trends in content selection and curriculum design

The data strongly supports the need for regular reflection on participants’ professional needs and preferences for curricular foci. This is evidenced by the frequent references to the themes of “competency-related curiosity” and “professional advancement”, along with the subthemes “chosen topics” and “practical relevance”. The profile of a curriculum and it’s content determines whether postgraduate students enrol for a study program [33]. Studies confirm that student satisfaction increases throughout the program if the courses and content are perceived as professionally directly relevant and interesting cf [54], [55], [56]. 

##### Implication 2: Offer content and tools with a high practical relevance

The emphasis on practical relevance, as reflected in the subthemes “interest in topic”, and “adapting to workplace changes” highlights a clear expectation for tools that can be directly applied in the workplace. Practical and work-related learning content influences professionals’ decisions to participate in a postgraduate education program [22], [29]. We imply that a strong alignment between the perceived professional relevance of a postgraduate education program and participants’ experiences in the program will lead to high satisfaction among participants.

##### Implication 3: Foster the program participants to be inspired by the exchange with other participants and faculty and thus build up networks

The role of networking emerged as a crucial factor, as seen in the themes “networking” and “network building”. In the postgraduate education domain, the influence of exchange between students and resulting satisfaction is hardly addressed in research. In undergraduate education, however, numerous studies confirm a direct correlation between social connectedness and satisfaction with the program. Interactions, dialogue with peers in the classroom, and integrating professional problems into learning experiences strengthen motivation, self-efficacy, and learning experiences. The exchange is perceived as relevant for lifelong learning and professional success [57], [58], [59]. 

##### Implication 4: Give the participants the knowledge and skills to gain “empowerment” in their work environment

Participants identified the theme of “empowerment” as a strong motivation for enrolling in postgraduate education programs, particularly the desire to “evoke change” within their organizations. The opportunity to influence changes and aspects of the work situation is of high importance for health professionals [49]. Competences related to changes are described in the literature about leadership competencies of physicians. Skills such as strategic and tactical planning, persuasive communication, negotiation and dialogue are required for advocacy and management roles to transform and empower the local environment [60]. 

##### Implication 5: Structure and organize the program curriculum by considering the needs of the target groups who attend the programs in an extra-occupational manner

The subtheme “structure and organization” in the sense of completing continuing education alongside work and family is of very high relevance. A program that can balance study time and personal and professional commitments is highly attractive to postgraduate students [61]. Thus, the structure and organization of a program are highly relevant to postgraduate students’ satisfaction [62].

##### Implication 6: Offer course formats and locations “close to home” 

The subtheme “proximity” highlights the importance of geographic accessibility. The significance of these themes is supported by existing literature, which indicates that the location of higher education institutions (HEIs) significantly influences postgraduate students’ decisions [63]. However, the importance of the location has only a relative relevance in this context because universities have a fixed location. One way to reduce the negative impact of distance is to offer hybrid teaching, allowing some part of the course participation to be attended at a distance. On the other hand, students appreciate the personal exchange on-site; it facilitates learning and enables local networking. 

##### Implication 7: Engage a highly qualified faculty

Participants highlighted the importance of “experts” competency, underscoring the critical role that high-quality teaching staff play in postgraduate education. Various studies with postgraduate students confirm the connection between the qualifications of the faculty and overall satisfaction with the education program [64], [65]. The quality of teaching staff has been identified as the highest performance gap in relation to student expectations, emphasizing the need for universities to prioritise hiring experienced and innovative lecturers [65].

### 5.3. Strengths and limitations

We conclude that integrating participants’ motivation to attend a program into program planning is a pertinent source of information to increase student satisfaction, offer high-quality, innovative teaching and learning opportunities, and keeping up with the competitiveness of HEI programs. The main motivation factors identified in the interviews align with those from the EPS [14], highlighted in the literature review, confirming their relevance to postgraduate education and also to other healthcare professions. The qualitative approach of this study also identified new inductive factors for consideration in program planning.

However, some limitations of this study must be acknowledged. 

First, with a sample size of only two postgraduate programs, it can be argued that our findings may not be generalisable to other postgraduate programs in the healthcare sector. Nevertheless, we maintain that the MAS MME, although focused on medical education, addresses needs that are broadly relevant to many healthcare professionals. This program is offered to an interdisciplinary and interprofessional audience, all of whom share the common role of teaching in their respective fields. To provide contrast, we also included a CAS program targeting clinical professionals in palliative care. Despite the distinct focus of these two programs, our results showed a striking convergence in motivation themes. The strong alignment between the identified themes and established theory, validated across diverse target groups, suggests that these findings may also be applicable to other healthcare professions.

Second, the relatively small sample size of 23 participants may be criticized as insufficient to support our findings. This is a common critique of qualitative studies. However as noted in the method part, we continued conducting interviews until we reached data saturation. Therefore, we argue that the number of interviewees was adequate to confirm the theoretically derived motivation criteria

Third, it could be argued that this qualitative study does not provide sufficient evidence to formulate implications for program planning. While this critique indeed is valid, we do not claim that our implications related to RQ3 are definitive. However, given that we found supporting literature for the defined implications, we believe they offer valuable insights and warrant further reflection. Furthermore, the practical implications of our findings, including their operationalisation and feasibility, can be further investigated in future studies.

The aforementioned limitations, particularly the limited number of programs studied and the relatively small participant sample, may restrict the generalizability of our results. These constraints affect the interpretation of our findings by suggesting that the identified motivational factors might not be universally applicable to all postgraduate programs within the healthcare sector. Future research could benefit from including a larger and more diverse range of programs to enhance the robustness and generalizability of the results. Additionally, employing a mixed-methods approach, which integrates both qualitative and quantitative data, could help validate the observed relationships and provide a more comprehensive understanding of the motivational factors at play.

## 6. Conclusion

We explored the motivation of healthcare professionals to participate in postgraduate education programs. The findings related to significant motivational factors, reveal significant convergence with theoretical constructs from the literature, as well as some divergences between different study groups. Overall, the high alignment between the interview data and established motivational factors extends the relevance of our results to various healthcare professions. 

Based on the identified motivational factors, we have suggested implications for program planning. These implications offer suggestions to better align programs with participants' needs, thereby increasing the likelihood of student retention, enhancing learning outcomes, and improving overall program effectiveness. Considering our presented insights in program development, we believe that student satisfaction can be improved. This underscores the need for providers to offer high-quality, innovative teaching and learning opportunities that align with participants’ needs and interests.

## Authors’ ORCIDs

- Melanie de la Rosa: [0009-0009-9169-0273]
- Felix M. Schmitz: [0000-0001-7755-4616]
- Joana Berger-Estilita: [0000-0002-8695-4264]
- Ara Tekian: [0000-0002-9252-1588]
- Sissel Guttormsen: [0000-0001-9932-1872]

## Acknowledgements

We thank all the participants who shared their valuable insights for this study. Their contributions have significantly enhanced our research. 

## Competing interests

The authors declare that they have no competing interests. 

## Supplementary Material

Question route

General motivation themes to participate in postgraduate education programs and sample quotes from the most frequently mentioned subthemes

Motivation themes to join a particular training program and sample quotes from the most frequently mentioned subthemes

## Figures and Tables

**Table 1:**
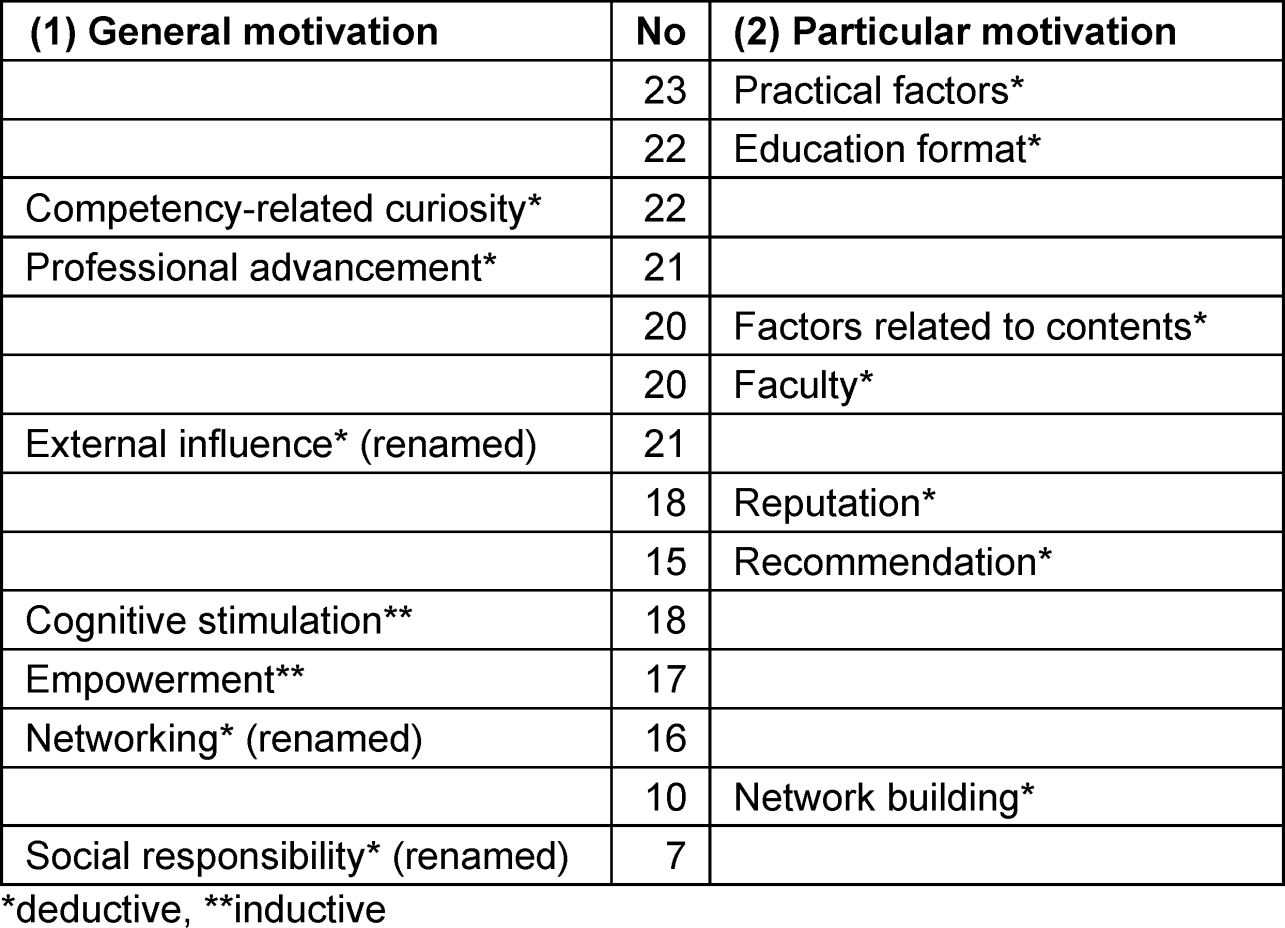
Overview general and particular motivation themes, sorted by mentions

**Table 2:**
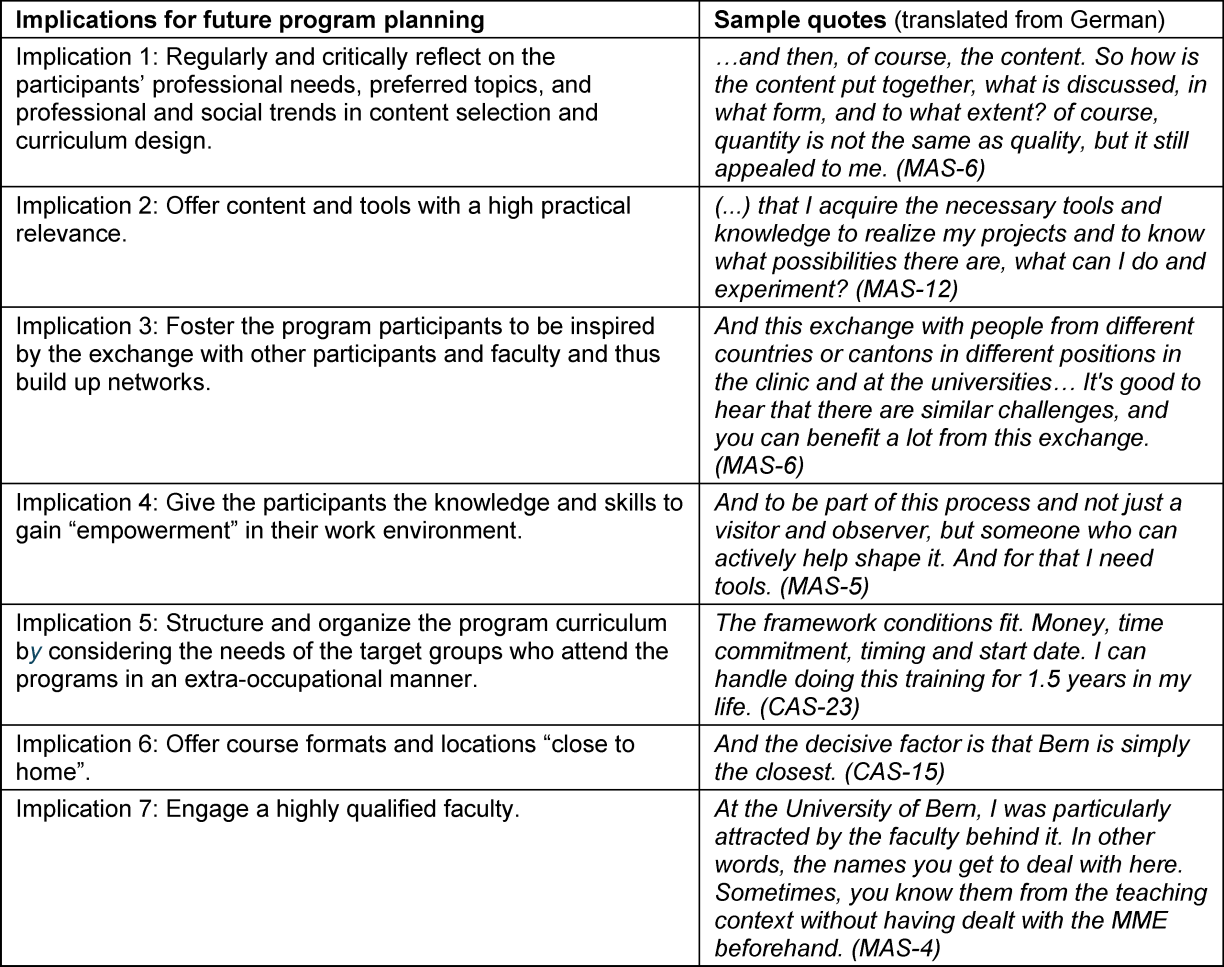
Implications for future program planning and sample quotes
