# Supplementary material for: Exploring healthcare professionals’ motivation to attend two postgraduate education programs at the University of Bern in Switzerland: A qualitative interview study
Source: GMS J Med Educ. 2025 Jun 16;42(3):Doc37. doi: 10.3205/zma001761 (PMC12286871; doi:10.3205/zma001761)
Supplement: Question route [file JME-42-37-s-001.pdf]

Attachment 1: Question route

1. Introduction to the interview

The participants were welcomed and thanked for their participation. After the interviewer briefly introduced herself and initial insights into the study topic, warm-up questions were asked:

a. What profession do you practice?
b. Why did you choose this profession/specialisation?
c. What do you enjoy most about your job?

2A. Questions related to research question 1: What motivates healthcare professionals to participate in postgraduate education programs in general?

The developed questions aim at investigating the following factors, identified in the literature review: *interpersonal relations, compliance with external influence, professional advancement, escape from routine, competency-related curiosity, community service*. The questions were by purpose formulated in an open way, to avoid emphasizing certain factors. The named theoretical factors were used for the deductive coding of the answers.

a. Opening question: What is your personal motivation for continuing your education with an MAS/CAS?
b. What should be different for you after the MAS/CAS than before?
c. How do you think (or expect) the MAS/CAS will have impact on you?
d. What will change in your life situation from attending this program?
e. What was the decisive reason to attend this program?
f. Does the MAS/CAS have any other relevance for you?
g. If you could wish for something professionally (or privately), what would it be?
h. What would your partner, your family or a good friend say if I asked them what motivates you to take part in this MAS/CAS?
i. If you had all the options, no restrictions or obstacles to your (professional) path, what would you then do?
j. Which circumstances in your private and/or professional life has led you to take part in the MAS/CAS right now? (If only intrinsic motivational factors are mentioned)
k. Were there other, externally driven reasons, i.e. influences coming from other people or the environment, which also contributed to start the MAS/CAS? (If only extrinsic motivational factors are mentioned)
l. What personal reasons are there, for instance personal development or personal needs to increase your satisfaction, that motivate you to start the MAS/CAS?
m. Can you think of anything else related to your personal motivation to take part in the MAS/CAS, that you want to share with me?

2B. Questions related to research question 2: What motivation factors influence the decision of health care professionals to attend a particular postgraduate education program?

The developed questions aim at investigating the following factors, defined in the literature review: *practical factors, factors related to contents, education format, network building, reputation, recommendation, faculty*. The rationale behind the open formulations is the same as described above.

a. Opening question: What particularly attracted you to this MAS/CAS program at the University of Bern?
b. Why did you choose a program at the university and not, for example, at a private institution?
c. Why did you choose the specialization Master of Medical Education/CAS Palliative?
d. Why do you want to attend a degree program with the title Master/CAS?
e. Have you also taken other programs into consideration? (including programs in other countries?)
f. What was crucial for your choice? What exactly makes this MAS/CAS program attractive to you?
g. To what extent does the chosen program fit in with your current life situation? …in very practical terms.
h. What are the biggest organizational challenges you face when participating in the MAS/CAS?
i. Which importance does the quality of the program has for you?
j. Can you think of anything else related to "Particular factors that influenced your decision" to attend this program, that I have not yet addressed?

3. Conclusion of the interview

The participant was thanked for participating in the interview. Any remaining open questions from the participants were answered. It was emphasized that the anonymised data would only be used in the context of the study. This was already confirmed by the participants in the previously sent consent letter. We offered to send the published article with the results of the interviews to the participants, which they appreciated and agreed to.
