# Supplementary material for: Exploring healthcare professionals’ motivation to attend two postgraduate education programs at the University of Bern in Switzerland: A qualitative interview study
Source: GMS J Med Educ. 2025 Jun 16;42(3):Doc37. doi: 10.3205/zma001761 (PMC12286871; doi:10.3205/zma001761)
Supplement: General motivation themes to participate in postgraduate education programs and sample quotes from the most frequently mentioned subthemes [file JME-42-37-s-002.pdf]

Attachment 2: General motivation themes to participate in postgraduate education programs and sample quotes from the most frequently mentioned subthemes

**General motivation main theme**<br>**General motivation subtheme (most mentioned)**	***Diff. MAS***	***Diff. CAS***	***Sample quotes***<br>*(translated from German)*
**Competency-related curiosity**<br>(n_{MAS}=13, n_{CAS}=9, Total=22)			
Interest in topic<br>(n_{MAS}=13, n_{CAS}=9, Total=22)	↑4		*And then you go into the literature a bit, and the MAS's offer covers exactly this toolbox, a word that I like quite a bit. (MAS-5)*
Personal development<br>(n_{MAS}=11, n_{CAS}=4, Total=15)	↑7		*Yes, there is a huge stimulus to keep learning, to keep developing and yes, I think that's really cool. (MAS-9)*
Specialization<br>(n_{MAS}=9, n_{CAS}=4, Total=13)	↑5		*Thus, my personal motivation is actually to specialize and to be able to identify myself as a specialist in the field of Palliative Care (CAS-17)*
Confirmation of own professional behaviour (n_{MAS}=4, n_{CAS}=1, Total=5)	↑3		*Perhaps even more a confirmation of what I have already done. (CAS-19)*
**Professional advancement**<br>(n_{MAS}=13, n_{CAS}=8, Total=21)			
Adapting to workplace changes<br>(n_{MAS}=12, n_{CAS}=6, Total=18)	↑6		*There was also a personal desire, from a professional background, to do something else, something completely different. (CAS-22)*
Career springboard<br>(n_{MAS}=9, n_{CAS}=6, Total=15)	↑3		*…that having the MAS gives you an advantage in your career over someone without the MAS. (MAS-2)*
Securing professional attractivity<br>(n_{MAS}=6, n_{CAS}=3, Total=9)	↑3		*Something that I can use in my field. Something that perhaps sets me apart a little from others, like having my own niche for the future. (CAS-14)*
Be a multiplier<br>(n_{MAS}=6, n_{CAS}=1, Total=7)	↑5		*And it fits perfectly right now that I am qualifying myself and can contribute input to the team to ensure the curriculum development goes well (MAS-10)*
**External influence (renamed)**<br>(n_{MAS}=13, n_{CAS}=8, Total=21)			
Manager as initiator<br>(n_{MAS}=9, n_{CAS}=1, Total=10)	↑8		*Yes, for me at the beginning, it was almost only external motivation that my boss said: wouldn't that be something for you? (MAS-9)*
Circumstances<br>(n_{MAS}=4, n_{CAS}=5, Total=9)		↑1	*So somehow it all fit together. The new position on the palliative care ward with 80% and the start of the course. (CAS-23)*
Family, colleagues, and friends as motivators (n_{MAS}=5, n_{CAS}=2, Total=7)	↑3		*Well, through some of my colleagues who have also done it, who then approached me, saying, "Look, you have been teaching for so long. Why don't you take the course?" (MAS-8)*
Role models as motivators<br>(n_{MAS}=4, n_{CAS}=1, Total=5)	↑3		*And then I also looked up the websites of various clinics and saw that there are a few senior doctors and chiefs who have done it, some of whom I knew from working together and thought they were great people. That's how I want to be, too.(MAS-2)*

Attachment 2 to De la Rosa M, Schmitz FM, Berger-Estilita J, Tekian A, Guttormsen S. *Exploring healthcare professionals' motivation to attend two postgraduate education programs at the University of Bern in Switzerland: A qualitative interview study*. GMS J Med Educ. 2025;42(3):Doc37. DOI: 10.3205/zma001761

Institutional obligation (n_{MAS}=1, n_{CAS}=4, Total=5)		↑3	*To specialize in palliative care in [country], you need to complete the CAS. (CAS-20)*
**Cognitive stimulation (Inductive)** (n_{MAS}=11, n_{CAS}=7, Total=18)			
Stimulation (n_{MAS}=7, n_{CAS}=3, Total=10)	↑4		*… when you've been doing this for over 20 years, it feels good to get a new intellectual input, to have new people, other people not from my area around me, with different perspectives (MAS-8)*
Fun and joy in learning (n_{MAS}=5, n_{CAS}=4, Total=9)	↑1		*…and it`s fun to learn (CAS-15)*
Passion for domain (n_{MAS}=3, n_{CAS}=2, Total=5)	↑1		*Perhaps it is because it was a matter of the heart. And what about pursuing further education or additional training in this field.(CAS-19)*
**Empowerment (Inductive)** (n_{MAS}=11, n_{CAS}=6, Total=17)			
Evoking changes (n_{MAS}=10, n_{CAS}=5, Total=15)	↑5		*In terms of the people I will train, I mean to educate them, but also help me to negotiate with the superiors, for example, with the organizations and say, yes, now we need to put more resources into this area, more people, more premises. Here are the numbers, that's what you need. (MAS-2)*
Carrying the title (n_{MAS}=6, n_{CAS}=3, Total=9)		↑7	*And I think the main reason was that I really wanted a recognized diploma. (CAS-21)*
Standing (n_{MAS}=6, n_{CAS}=1, Total=7)	↑5		*So, I think in the academic world, and you can think that's good or bad, but a lot of it is about the titles and that you somehow have a track record and can say, yes, I have completed this program and that is then really also proof that one knows the topic of medicine didactics. (MAS-6)*
**Networking (renamed)** (n_{MAS}=10, n_{CAS}=6, Total=16)			
Inspiration through exchange (n_{MAS}=9, n_{CAS}=5, Total=14)	↑4		*I think the main reason was actually also the interprofessional exchange because I think the professions in particular could perhaps benefit from each other even more for the benefit of the clients. (CAS-21)*
Build a network (n_{MAS}=9, n_{CAS}=3, Total=12)	↑6		*…and to build up a good network in the program as well with colleagues. (MAS-1)*
**Social responsibility (renamed)** (n_{MAS}=4, n_{CAS}=3, Total=7)			
	↑1		*The work with patients will also benefit, …, a lot of new information is coming in. That's what it's going to be about. So, for me, it's about the patient benefiting, that I work for him. (CAS-15)*

Legend: Diff.= Differences, ↑ more mentions in direct group comparison
