# Supplementary material for: Exploring healthcare professionals’ motivation to attend two postgraduate education programs at the University of Bern in Switzerland: A qualitative interview study
Source: GMS J Med Educ. 2025 Jun 16;42(3):Doc37. doi: 10.3205/zma001761 (PMC12286871; doi:10.3205/zma001761)
Supplement: Motivation themes to join a particular training program and sample quotes from the most frequently mentioned subthemes [file JME-42-37-s-003.pdf]

Attachment 3: Motivation themes to join a particular training program and sample quotes from the most frequently mentioned subthemes

**Main motivation theme particular training**<br>Subtheme particular training (most mentioned)	***Diff. MAS***	***Diff. CAS***	***Sample quotes***<br>*(translated from German)*
**Practical factors**<br>(n_{MAS}=13, n_{CAS}=10, Total=23)			
Proximity<br>(n_{MAS}=11, n_{CAS}=9, Total=20)	↑2		*So certainly, the fact that it's localized in Bern. That just makes it logistically much easier for me. (MAS-8)*
Support from employer<br>(n_{MAS}=7, n_{CAS}=4, Total=11)	↑3		*…part of the training is co-financed by the hospital. So not only the fees themselves but also part of the time. (CAS-17)*
**Education format**<br>(n_{MAS}=13, n_{CAS}=9, Total=22)			
Structure and organization<br>(n_{MAS}=13, n_{CAS}=7, Total=20)	↑6		*And I saw. This is well compatible with my professional life because there are only a few days that I am absent from the clinic and the final thesis and all that, which we still have to do in parallel and would be feasible. Besides the job and the family. (CAS-14)*
Method<br>(n_{MAS}=4, n_{CAS}=5, Total=9)		↑1	*And there is also a lot of exchange. Much of the work is done in small groups. We submit preliminary work, and then a case you might have submitted is discussed (CAS-15)*
**Factors related to contents**<br>(n_{MAS}=12, n_{CAS}=8, Total=20)			
Chosen Topics<br>(n_{MAS}=9, n_{CAS}=6, Total=15)	↑3		*And all the courses appealed to me in terms of the topics. But I finally attended the course because there is something from all topics and not only one topic discussed in detail. The entire knowledge is expanded in relation to different levels. (CAS-16)*
Practical relevance<br>(n_{MAS}=10, n_{CAS}=2, Total=12)	↑8		*So, I think practically in everyday professional life it fits perfectly, when I look at the topics, then they are exactly the things that occupy me. (MAS-6)*
One stop shop<br>(n_{MAS}=3, n_{CAS}=3, Total=6)			*The quality is that, within a manageable time frame, you actually get to relevant topics in Medical Education, not just a superficial glance, but also a deeper look beneath the surface. (MAS-5)*
Evidence based<br>(n_{MAS}=5, n_{CAS}=0, Total=5)	↑5		*… and there were a few keywords, such as evidence-based teaching methods, innovative teaching projects, medical didactic research, those were the keywords. (MAS-1)*
**Faculty**<br>(n_{MAS}=12, n_{CAS}=8, Total=20)			
Experts Competency<br>(n_{MAS} =9, n_{CAS}=8, Total=17)	↑1		*In summary, one of the main reasons, after studying the program, was really the personalities that help shape the MAS. (MAS-5)*

Attachment 3 to De la Rosa M, Schmitz FM, Berger-Estilita J, Tekian A, Guttormsen S. *Exploring healthcare professionals' motivation to attend two postgraduate education programs at the University of Bern in Switzerland: A qualitative interview study*. GMS J Med Educ. 2025;42(3):Doc37. DOI: 10.3205/zma001761

International perspectives (n_{MAS}=5, n_{CAS}=0, Total=5)	↑5		*… because I believe it provides the perspective of getting to know other healthcare and education systems. This international expertise, with renowned experts teaching, is something I found very important. (MAS-6)*
**Reputation** (n_{MAS}=12, n_{CAS}=6, Total=18)			
Reputation organization (MAS=10, n_{CAS}=5, Total=15)	↑5		*So somewhere I had, I don't know why, but a good feeling. The University of Bern has a good reputation for me. I don't know why whether that is justified or not. And a lot of doctors with us come from Bern and did their further and advanced training there and it feels good… (MAS-9)*
Reputation programme (n_{MAS}=9, n_{CAS}=3, Total=12)	↑6		*I know several people who have completed the MME program, and they were all enthusiastic about it. These are people from various fields, some still working at the university, some in other hospitals, both in clinical and non-clinical roles. I have heard nothing but positive feedback, not just for the title or the MME stamp, but for the high quality of the content. (MAS-8)*
**Recommendation** (n_{MAS}=10, n_{CAS}=5, Total=15)			
Influence of peer (n_{MAS}=9, n_{CAS}=3, Total=12)	↑6		*Yes, perhaps it is simply the colleague who did it before me. The exchange with her, I think, was the deciding factor. Definitely. (CAS-15)*
Influence of leadership/role model (n_{MAS}=6, n_{CAS}=3, Total=9)	↑3		*Hmm, I have to be honest, I didn't research it too thoroughly because it was so strongly recommended by my boss. (MAS-11)*
**Network building** (n_{MAS}=6, n_{CAS}=4, Total=10)			
	↑2		*Those were the keywords – the network building and network – that appealed to me the most, …. (MAS-1)*

Legend: Diff.= Differences, ↑ more mentions in direct group comparison
